# Morphological abnormalities in gall-forming aphids in a radiation-contaminated area near Fukushima Daiichi: selective impact of fallout?

**DOI:** 10.1002/ece3.949

**Published:** 2014-01-13

**Authors:** Shin-ichi Akimoto

**Affiliations:** Department of Ecology and Systematics, Graduate School of Agriculture, Hokkaido UniversityKita-ku, Sapporo, 060-8589, Japan

**Keywords:** malformation, morphology, radiation, *Tetraneura*, viability

## Abstract

To evaluate the impact of fallout from the Fukushima Daiichi Nuclear Power Plant accident on organisms, this study compared the morphology and viability of gall-forming aphids between the Fukushima population and control populations from noncontaminated areas. This study, in particular, focused on the morphology of first-instar gall formers derived from the first sexual reproduction after the accident. Of 164 first instars from *Tetraneura sorini* galls collected 32 km from Fukushima Daiichi in spring 2012, 13.2% exhibited morphological abnormalities, including four conspicuously malformed individuals (2.4%). In contrast, in seven control areas, first instars with abnormal morphology accounted for 0.0–5.1% (on average, 3.8%). The proportions of abnormalities and mortality were significantly higher in Fukushima than in the control areas. Similarly, of 134 first instars from *T. nigriabdominalis* galls, 5.9% exhibited morphological abnormalities, with one highly malformed individual. However, of 543 second-generation larvae produced in *T. sorini* galls, only 0.37% had abnormalities, suggesting that abnormalities found in the first generation were not inherited by the next generation. Although investigation is limited to one study site, this result suggests that radioactive contamination had deleterious effects on embryogenesis in eggs deposited on the bark surface, but a negligible influence on the second generation produced in closed galls. Furthermore, analysis of both species samples collected in spring 2013 indicated that the viability and healthiness of the aphids were significantly improved compared to those in the 2012 samples. Thus, the results of this study suggest the possibility that a reduced level of radiation and/or selection for radiation tolerance may have led to the improved viability and healthiness of the Fukushima population.

## Introduction

There is increasing interest in the effects of radionuclides released from nuclear power plant accidents on the biodiversity and genetics of wildlife (Møller and Mousseau 2006; Yablokov et al. 2009; Mousseau and Møller 2012a; Møller et al. 2013). Even 20 years after the 1986 Chernobyl nuclear disaster, bird and arthropod species abundances are negatively correlated with the level of radioactivity at ground level (Møller and Mousseau 2007, 2009). It is reasonable to suppose that the largest impact on biodiversity occurs immediately after an accident through the large amount of fallout. Alternatively, it is also possible that mutations accumulate over time and express deleterious effects several years after the accident in contaminated areas (Møller and Mousseau 2006). At present, we have limited knowledge about the effects of radiation on the wildlife. In the case of the Chernobyl nuclear disaster, the initial impacts on the abundance, phenotypes, and genetics of animal and plant species were not fully assessed (Yablokov et al. 2009; Møller et al. 2013). The Fukushima Daiichi Nuclear Power Plant exploded in mid-March 2011 and released 11,347 PBq of radionuclides, including 160 PBq of iodine-131, 15 PBq of cesium-137, and 0.14 PBq of strontium-90 into the atmosphere (Nuclear Emergency Response Headquarters, Government of Japan. 2011). An area northwest of the power plant was covered with fallout and highly contaminated by radioactive substances (Nuclear Regulation Authority, 2011a). For the Fukushima Daiichi accident, a vast area of contamination, including several landscapes, can be surveyed without legal restrictions, except within a 20-km radius of the power plant, and thus, there are opportunities to detect initial impacts of radionuclides on individual species and the ecosystem. Therefore, ecological and genetic investigations in the Fukushima area are an urgent issue.

Several researchers and governmental agencies have started evaluating the levels of radionuclides accumulated in wild animals and plants in the Fukushima area (e.g., Nakanishi and Tanoi 2013). Ishida (2013) started a long-term survey on the abundance of birds and mammals and confirmed a high level of radioactive contamination in bird feathers in the first year. Møller et al. (2012, 2013) conducted field censuses of birds and several insects and reported a significant negative correlation between densities and background radiation level. Hiyama et al. (2012) reported that the lycaenid butterfly *Zizeeria maha* that emerged in 2011 in contaminated areas exhibited morphological abnormalities and that these abnormalities were inherited by the next generation, which exhibited a higher proportion of abnormalities than the first generation. Hiyama et al.'s report is the first example of phenotypic abnormalities in the Fukushima area, so it is difficult to understand whether the occurrence of phenotypic abnormalities is common to the populations of other animals and plants in the Fukushima area or not. Thus, phenotypic and genetic studies of other systems might be encouraged.

In attempts to clarify the effects of radiation on mammals, birds, and arthropods, phenotypic abnormalities have been evaluated based on adults (Møller 1993, 2002; Møller and Mousseau 2006; Møller et al. 2007; Yablokov et al. 2009) except in a few studies (Williams et al. 2001). However, the effects of radiation emerge most strongly in hatchlings or newborns, resulting in mortality and phenotypic abnormalities, as shown in irradiation experiments (Elbadry 1965; Tilton et al. 1966; McGregor and Newcombe 1968; Vereecke and Pelerents 1969; Matranga et al. 2010) as well as field studies in Chernobyl (Krivolutzkii and Pokarzhevskii 1992; Krivolutsky 1995). When exposed to high radiation, hatchlings or newborns often fail to grow, with few individuals reaching adulthood. Therefore, it is possible to overlook the emergence of abnormalities if the research focus is limited to adult phenotypes only (Møller 1997). The present study focuses on gall-forming aphids and reports the extent to which morphological abnormalities emerge in first-instar aphids derived from the first sexual reproduction after the Fukushima Daiichi nuclear accident.

Aphids are tiny, soft-bodied insects that reproduce asexually from spring to autumn, with many consecutive generations (Dixon 1998). The use of aphids for a bioassay of radiation appears to have three merits. First, aphids always maintain high reproduction rates, with numerous developing embryos in their abdomens (Dixon 1998). Reports indicate that rapidly developing embryos are readily affected by radiation (Russell and Russell 1952; Vereecke and Pelerents 1969; Cerutti 1974). After the fallout that occurred in mid-March 2011, aphids continued to reproduce asexually under radioactive contamination until autumn, so that aphid embryos likely were damaged by radiation. Genetic damages, if any, would be inherited and accumulated through clonal lines. Through sexual reproduction in autumn 2011, damaged genes may have been genetically recombined to the next generation, leading to a release of a large phenotypic variation (Lynch and Gabriel 1983). Second, as hemimetabolous insects, aphid first instars possess several metrical characters, which would facilitate the detection of morphological abnormalities. So far, aphid first instars have been reported to exhibit phenological and morphological traits that well reflect genetic and environmental variation (Akimoto 1988, 1990, 1998, 2006; Komatsu and Akimoto 1995; Akimoto and Yamaguchi 1997; Komazaki 1998). Third, in gall-forming aphids, first-instar larvae induce leaf galls, in which they develop and produce offspring (Blackman and Eastop 1994). Thus, using aphid species that induce closed galls, the performance and mortality of gall formers can be evaluated without the disturbance of predators (Whitham 1978; Akimoto and Yamaguchi 1994; Wool 2004; Aoyama et al. 2012). If gall formers die in the gall, we can examine the morphology, and if they show some deformation, we can exclude the possibility of predation as a cause (Ballengée and Sessions 2009; Bowerman et al. 2010). The presence of malformed first instars in gall-forming aphids has been reported previously (Akimoto 1985a).

In the present study, I collected closed galls of *Tetraneura* aphid species from leaves of *Ulmus davidiana* var. *japonica* at a point 32 km from the Fukushima Daiichi Nuclear Power Plant and inspected the morphology of the gall formers at the first stadium. I report frequent occurrences of morphological abnormalities and mortalities in two gall-forming aphids in the Fukushima area by comparing them with other populations in noncontaminated areas. Although investigation is limited to one site in Fukushima, the results from two species corroborate the possibility of an initial, detrimental impact of fallout.

## Materials and Methods

### Life cycle of *Tetraneura* aphids

*Tetraneura* species are host-alternating and associated with two kinds of plants: elm trees *Ulmus* spp. as the primary host and gramineous plants as the secondary host (Blackman and Eastop 1994; Wool 2004). In spring, after hatching from overwintered eggs on the trunks of elm trees, first instars move to developing new leaves and start gall formation. The first instar (gall former) settles on the underside of a new leaf and, using its stylet, stimulates one point of the leaf. The stimulated plant tissues rapidly proliferate upward, encasing the gall former inside, to form a closed, hollow gall. During gall formation, the gall formers continuously stimulate the tissues without moving. The gall former develops inside the gall and parthenogenetically produces second-generation larvae (Fig. S1). The second generations develop into winged adults, which emerge from the cracked galls in mid-June and migrate to gramineous plants to produce third-generation larvae. These larvae move to the roots, where they develop into wingless adults. On the roots, several generations of wingless adults repeat reproduction from early summer until autumn, when winged adults emerge and return to elm trees. Winged adults produce wingless males and sexual females on the trunks of elm trees. After copulation, the sexual female oviposits a single egg in a crevice of the bark. Eggs overwinter until the next spring.

### Gall collection in Fukushima

I collected a total of 284 galls of *Tetraneura* aphid species from 1 *U. davidiana* var. *japonica* tree at Kawamata Town, Fukushima Prefecture (37^o^ 35′ N, 140 ^o^ 42′ E) on 3 June 2012. Leaves with galls were haphazardly collected from the tree, and all galls on the collected leaves were preserved in 80% ethanol. Galls of *Tetraneura* species are bean or horn shaped, a maximum of 25 mm in height, and formed in various positions on a leaf. On one leaf, 10 or more galls of two or more species are sometimes formed. The sample aphids were collected on roadsides that are not included in special reservation areas, so that no specific permissions were required for the collections. The aphid species collected are not designated as endangered or protected species.

After fixation, all galls were dissected under a binocular microscope. When the gall former gave birth to larvae, the adult and the cast-off skin of the first-instar gall former were collected from the gall and mounted on a glass slide. In the Eriosomatinae, the cast-off skin of the first-instar gall former is strongly sclerotized and characterized by species-specific features (Aoki 1975; Akimoto 1983, 1985b). The second-generation larvae produced in a gall were preserved in a vial of 80% ethanol, and all the larvae were later inspected for morphological abnormality. When the gall former was immature, only the cast-off skin of the first instar was mounted on a glass slide. When the gall former died in the gall for some reason, it was mounted for morphological examination.

Before mounting, first-instars' cast-off skins and adult gall formers were kept in 10% KOH for 1 or 2 days. The exoskeleton of the adult or first instar was rinsed in 80% ethanol with a small amount of acetic acid and dehydrated in carboxylol (phenol:xylol = 4:1) for 15 min and then in xylol for 10 min. The dehydrated samples were mounted on a glass slide with Canada balsam. All of the mounted specimens are preserved in the Laboratory of Systematic Entomology, Graduate School of Agriculture, Hokkaido University. No permits were required for the described study, which complied with all relevant regulations.

### Gall collection in other areas

Prior to the Fukushima Daiichi nuclear accident, no specimens had been collected in or around the Fukushima area. However, numerous specimens of *Tetraneura* species were collected before the accident in several localities of Japan and are preserved in the Laboratory of Systematic Entomology, Graduate School of Agriculture, Hokkaido University (Table 1). These specimens were used for comparison with the Fukushima specimens. In 2012, I collected *Tetraneura* aphids in Kashiwa, Chiba Prefecture (35°53′N, 139°56′E), Sapporo, Hokkaido (43°4′N, 141°20′E), and Iwamizawa, Hokkaido (43°11′N, 141°46′E), as control specimens. The sample aphids were collected on public parks and a university campus that are not included in special reservation areas, so that no specific permissions were required for the collections.

**Table 1:** Collection data of *Tetraneura* samples used for the inspection of morphological abnormalities. Galls or larval gall formers of *Tetraneura sorini* or *T. nigriabdominalis* were collected from *Ulmus davidiana* or *U. parvifolia*

Species	Locality (Japan)	Year	Host tree	Stage	No.
*T. sorini*	Bibai, Hokkaido	5 June 1981	*U. davidiana*	Larva	334
*T. sorini*	Bibai, Hokkaido	22 May 1987	*U. davidiana*	Larva	385
*T. sorini*	Iwamizawa, Hokkaido	8 June 1997	*U. davidiana*	Gall	108
*T. sorini*	Iwamizawa, Hokkaido	9 June 2012	*U. davidiana*	Gall	290
*T. sorini*	Sapporo, Hokkaido	22 May 1989	*U. davidiana*	Larva	99
*T. sorini*	Kawamata, Fukushima	3 June 2012	*U. davidiana*	Gall	167
*T. sorini*	Kawamata, Fukushima	21 May 2013	*U. davidiana*	Gall	50
*T. sorini*	Kashiwa, Chiba	30 April 2012	*U. davidiana*	Larva	243
*T. sorini*	Ukiha, Fukuoka	8 April 1985	*U. parvifolia*	Larva	100
*T. nigriabdominalis*	Bibai, Hokkaido	5 June 1981	*U. davidiana*	Larva	112
*T. nigriabdominalis*	Sapporo, Hokkaido	5 June 2012	*U. davidiana*	Gall	224
*T. nigriabdominalis*	Kawamata, Fukushima	3 June 2012	*U. davidiana*	Gall	136
*T. nigriabdominalis*	Kawamata, Fukushima	21 May 2013	*U. davidiana*	Gall	347
*T. nigriabdominalis*	Kashiwa, Chiba	30 April 2012	*U. davidiana*	Larva	564
*T. nigriabdominalis*	Kyoto, Kyoto	25 April 1981	*U. davidiana*	Larva	454
*T. nigriabdominalis*	Tsu, Mie	16 May 1991	*U. davidiana*	Gall	95
			*U. parvifolia*	Gall	47
*T. nigriabdominalis*	Ukiha, Fukuoka	8 April 1985	*U. parvifolia*	Larva	181

Gall collection, the examination of the gall contents, and the preparation of slide-mounted specimens were conducted as in the Fukushima samples. Where first-instar larvae were sampled, I haphazardly collected developing host buds infested with the first instars and preserved them in vials of 80% ethanol. Later, first instars were removed from the buds and mounted on glass slides.

### Morphological abnormalities and mortality

All mounted specimens of two common species, *T. sorini* and *T. nigriabdominalis*, were examined under a microscope (Axiophoto, Carl Zeiss, Oberkochen, Germany) for the morphological abnormalities and mortality. Because first-instar gall formers from all the samples were available for the microscopic inspection, the present study focused specifically on comparisons of first-instar morphology; 167 *T. sorini* first instars and 136 *T. nigriabdominalis* first instars from the Fukushima area were compared with 1559 *T. sorini* first instars from seven control areas and 1677 *T. nigriabdominalis* first instars from six control areas, respectively. For all the mounted specimens, including dead individuals, I inspected the morphology of legs, antennae, rostra, and tergites. The first instars of *T. sorini* and *T. nigriabdominalis* were approximately 0.90 ± 0.099 (SD) mm and 0.72 ± 0.028 (SD) mm in body length, respectively.

Several morphological abnormalities, ranging from slight variants to serious deformations, were found among first-instar gall formers. The abnormalities were classified into three categories, and the proportion of each category was calculated for samples in each area. The levels of abnormalities were determined irrespective of mortality; that is, dead first instars may be considered normal morphologically, while first instars in any of three abnormality categories may molt to the next larval stadium. Categorization of abnormal first instars was conducted for demonstrating the peculiarity of the Fukushima population, but in statistical tests, first instars with any abnormal morphology were pooled. Mortality was assessed for each gall former at the time galls were collected.

### Comparison of growth performance

To examine whether growth performance in the Fukushima samples was reduced or not, the growth rate of gall formers was compared between the Fukushima and Iwamizawa samples collected in early June 2012. Based on the cast-off skin of the first instar and the adult collected from each gall, I measured the lengths of larval and adult hind femurs of the same individual using an eyepiece micrometer installed on the microscope. The regression line of adult femur length on larval femur length demonstrates the mode of growth in each population. A steeper slope of the regression line indicates higher growth performance. Thus, ANCOVA (Sokal and Rohlf 1995) was applied to detect the difference in the regression slope or the intercept between the two populations. Galls from Fukushima were collected from one host tree; galls from Iwamizawa were collected from three host trees and pooled in the analysis.

### Comparison between the 2012 and 2013 samples from Fukushima

To evaluate temporal changes in the viability and healthiness of the Fukushima population, *Tetraneura* galls were again collected from the same elm tree from which the 2012 sample was collected, on 21 May 2013. For the 2013 samples, 50 and 347 gall formers of *T. sorini* and *T. nigriabdominalis,* respectively, were mounted on glass slides. The proportions of morphological abnormality and mortality were evaluated based on the gall formers at the first to the last stadium. By sampling galls from the same tree in both years, it is possible to remove the effects of plant factors on the morphology and viability of gall formers.

Logistic regression was used to test whether the proportions of morphological abnormality and mortality changed or not between the 2 years. In the model, the proportions of healthy, abnormal, and dead gall formers were treated as the dependent variable, while species (*T. sorini* or *T. nigriabdominalis*) and years (2012 or 2013) were specified as independent variables. The interaction between years and species was also included in the model.

### Measurements of radioactivity

Radioactivity levels were measured at the time of gall collection using Geiger counters (SOEKS-01M [SOEKS, Russia] and PA-1000 Radi [Horiba, Kyoto]). Measurement was conducted at a height of 1 m and at the ground level near the tree from which galls were collected. For the other areas in 2012 (Kashiwa, Sapporo, and Iwamizawa), I used the published data from the document “Monitoring information of environmental radioactivity level” by the Nuclear Regulation Authority, Government of Japan (http://radioactivity.nsr.go.jp/map/ja/).

## Results

### Categorization of abnormalities

From the 284 galls collected in Fukushima, four species of gall-forming *Tetraneura* were found, including 162 *T. sorini*, 118 *T. nigriabdominalis*, 3 *T. radicicola*, and 1 undescribed species. Galls occasionally contained two or more gall formers inside (of the *T. sorini* and *T. nigriabdominalis* galls, 3.7% and 9.3%, respectively). From the *T. sorini* galls, 167 gall formers were found, and 76 (71.7% of gall formers that attained at least the second instar) gave birth to larvae. From the *T. nigriabdominalis* galls, 136 gall formers were found, and only 1 (2.2%) gave birth to larvae.

I detected several morphological abnormalities in the first-instar gall formers of the two species both from Fukushima and from the other areas. Slight abnormalities (level 1) included the atrophy or bending of 1 leg (Fig. S2A,B), small ganglia on the ventral surface (Fig. S2C), partial fusion of adjacent abdominal tergites (Fig. S2D), and tissue necrosis in 1 leg or antenna (Fig. S3A,B). Tissue necrosis that occurred inside an appendage resulted in the partial or complete loss of that appendage after molting. Level 2 abnormalities included the atrophy or bending of 2 legs and tissue necrosis in two or more appendages. I categorized the complete or partial loss of one appendage of first instars as level 2 (Fig. S3C,D). Such first instars appear to have already lost an appendage at their hatching. Intense level 3 abnormalities included the loss of two or more appendages, the loss of 1 leg and atrophy of another leg, the appearance of new features, and conspicuous asymmetry in bilateral characters. These abnormalities were common in the two species.

At the time of gall collection in Fukushima on 3 June 2012, the radiation dose was 4.0 *μ*Sv/h at 1 m and 6.0 *μ*Sv/h at ground level. In contrast, the radiation doses in Kashiwa, Sapporo, and Iwamizawa were 0.117, 0.038, and 0.040 *μ*Sv/h, respectively.

### Abnormalities and mortality in *T. sorini*

**Figure 1:**
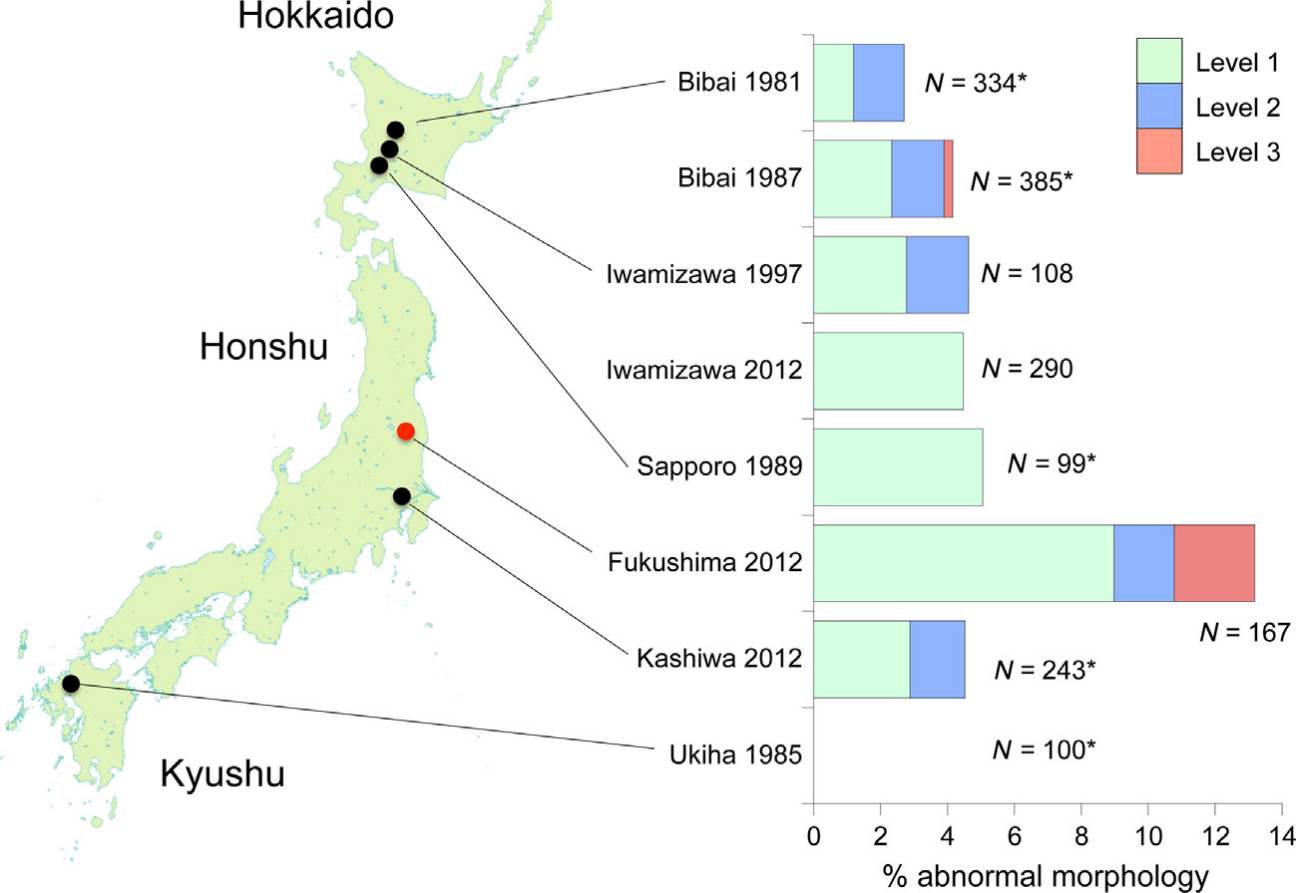
Percentage abnormal morphologies in *Tetraneura sorini* first-instar gall formers from eight populations. Asterisks in sample sizes indicate that first instars were collected from buds, whereas nonasterisked figures indicate that first-instars' cast-off skins were collected from galls. Level 1, slight abnormalities included the atrophy or bending of one leg (Fig. S2A,B), small ganglia on the ventral surface (Fig. S2C), partial fusion of adjacent abdominal tergites (Fig. S2D), and tissue necrosis in one leg or antenna (Fig. S3A,B). Level 2, abnormalities included the atrophy or bending of two legs and tissue necrosis in two or more appendages. I categorized the complete or partial loss of one appendage of first instars as level 2 (Fig. S3C,D). Level 3, intense abnormalities included the loss of two or more appendages, the loss of one leg and atrophy of another leg, the appearance of new features, and conspicuous asymmetry in bilateral characters.

**Figure 2:**
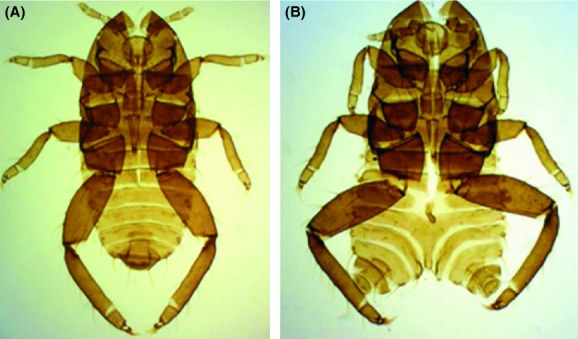
Cast-off skins of first-instar gall formers of *T. sorini* from Fukushima. (A) normal morphology, (B) level-3 malformation with a bifurcated abdomen.

**Figure 3:**
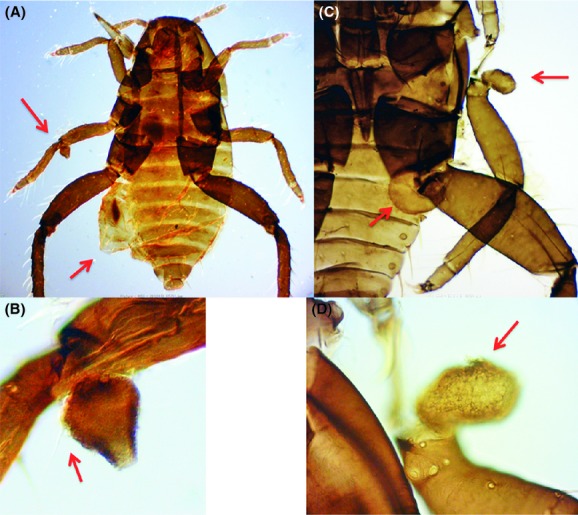
Two level-3 malformed *T. sorini* first instars from Fukushima. (A) a dead first instar having a distended abdomen (arrowed) and a projection on the joint of the mid-femur and tibia (arrowed). (B) projection having a seta (arrowed), (C) a dead first instar having protuberances on the abdomen (arrowed) and the base of a mid-leg (arrowed). The mid-leg was transposed from the original position. (D) protuberance on the base of the trochanter.

**Figure 4:**
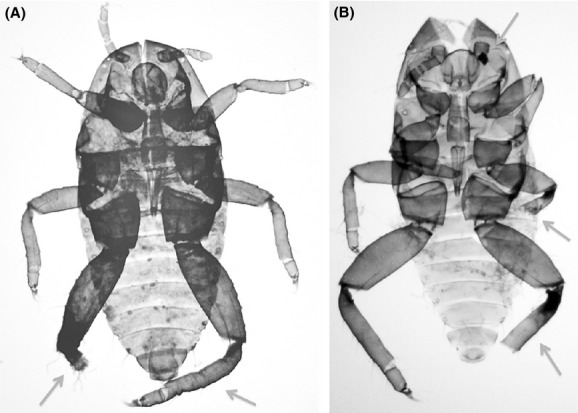
Two level-3 malformed first instars from Fukushima. (A) dead *T. sorini* first instar with a hind tibia missing due to tissue necrosis (arrowed) and the other hind leg atrophied (arrowed). (B) cast-off skin of a *T. nigriabdominalis* first instar with a hind leg, a mid-leg, and an antenna missing (arrowed).

Of the 167 *T. sorini* first instars collected in Fukushima, 13.2% exhibited any of the abnormalities (Fig. 1). Of the seven control areas, the samples from Ukiha, Fukuoka Prefecture, exhibited no abnormalities. Of the samples from Sapporo in 1990, 5.1% exhibited level-1 abnormalities, and this value was the maximum found in the control areas (Fig. 1). The incidence of morphological abnormalities in Fukushima was significantly higher than that in other areas (Table 2; on average, 3.8% for other areas). The Fukushima samples were peculiar because they contained four level-3 malformed individuals (Fig. 1). One of the individuals had a bifurcated abdomen with 2 caudae (Fig. 2). Despite this intense malformation, this individual molted successfully four times and attained adulthood with two caudae. Although larviposition was not confirmed, this individual contained mature embryos in its abdomen. The second example (Fig. 3A) was found dead in an incipient gall. This individual had an empty, distended abdomen and a projection on the joint of the mid-femur and tibia; this projection bore several setae, suggesting that the projection was a homologous leg structure. The third example (Fig. 3B) was also found dead in a gall and bore a large protuberance on the abdomen. In addition, it had another solid protuberance at the base of the mid-femur. The last example (Fig. 4A), which was also found dead in a gall, had 1 hind leg with apical segments missing due to necrosis and another hind leg that was atrophied.

**Table 2:** Comparison of the proportion of morphological abnormalities between the Fukushima population and control populations. *P-*values from log-likelihood ratio tests were adjusted for multiple comparisons using the sequential Bonferroni method

Comparison of populations		χ^2^	*P*
Fukushima 2012	Bibai 1981	19.61	<0.0001*
Fukushima 2012	Bibai 1987	13.44	0.0002*
Fukushima 2012	Iwamizawa 1979	5.41	0.0148*
Fukushima 2012	Iwamizawa 2012	10.81	0.001*
Fukushima 2012	Sapporo 1989	4.94	0.0262*
Fukushima 2012	Kashiwa 2012	9.83	0.0017*
Fukushima 2012	Ukiha 1985	21.82	<0.0001*

* Significant difference at *P *=* *0.05 after the application of the sequential Bonferroni method.

**Figure 5:**
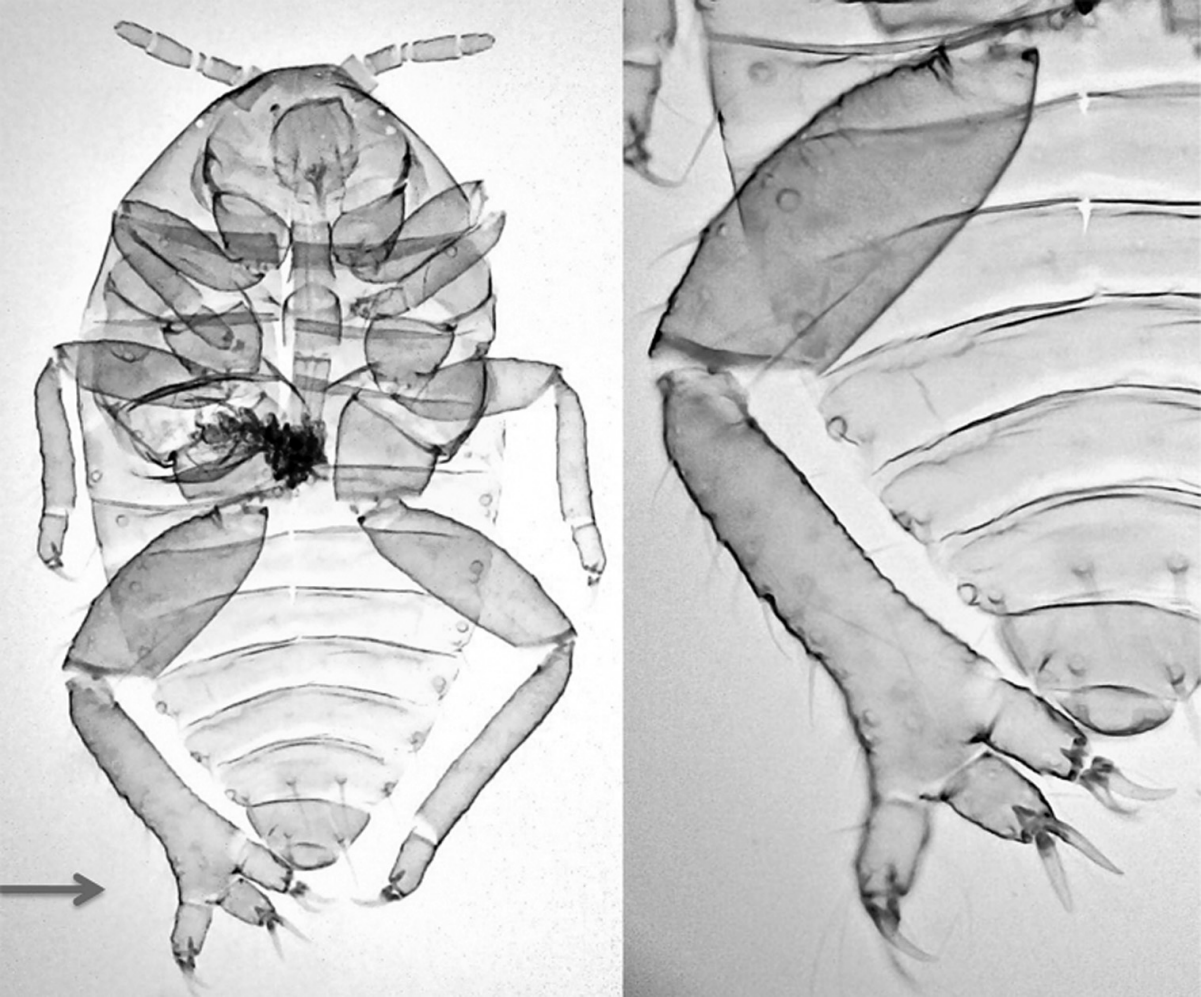
*T. sorini* first-instar gall former with a trifurcated tarsus (arrowed) collected from Bibai, Hokkaido in 1987.

In the areas other than Fukushima, only 1 *T. sorini* first instar exhibited a conspicuous level-3 malformation. This first instar, collected in Bibai City in 1987, had a trifurcated tarsus on 1 hind leg that was thicker than the other (Fig. 5). Therefore, except for Fukushima, a level-3 malformation was found only once in 1559 individuals (0.064%).

Mortality in the gall formers in the Fukushima population was compared with that in the Iwamizawa populations collected in 1997 and 2012 (Fig. S4A). Mortality in Fukushima was 14.4%, which was significantly higher than the mortality in Iwamizawa (1.9%–3.2%) (log-likelihood ratio test, for 1997 Iwamizawa, χ^2^ = 14.7, *P *=* *0.0001; for 2012 Iwamizawa, χ^2^ = 19.0, *P *<* *0.0001). To examine whether this difference was due to host suitability or not, the growth rate of gall formers was compared between the Fukushima and Iwamizawa samples collected in 2012. ANCOVA for the two populations indicated that there were no significant differences in the slopes or intercepts of the regression lines (Fig. S5; for area*larval femur length, *t* = −1.45, *P *=* *0.150; for area, *t* = 0.78, *P *=* *0.436; for larval femur length, *t* = 11.91, *P *<* *0.0001). This suggests that despite the difference in mortality, the growth performance of the gall formers that survived the larval stages did not vary between the two populations.

I inspected a total of 543 second-generation larvae deposited in 76 galls collected in Fukushima. Only 2 larvae (0.37%) from 1 gall (that is, they are clonal) were found to have aberrant morphology; they both lacked 1 antenna completely, whereas the other larvae in the same gall were normal. The gall former in that gall did not exhibit abnormality at the first stadium or adulthood.

### Abnormalities in *T. nigriabdominalis* and *T. radicicola*

**Figure 6:**
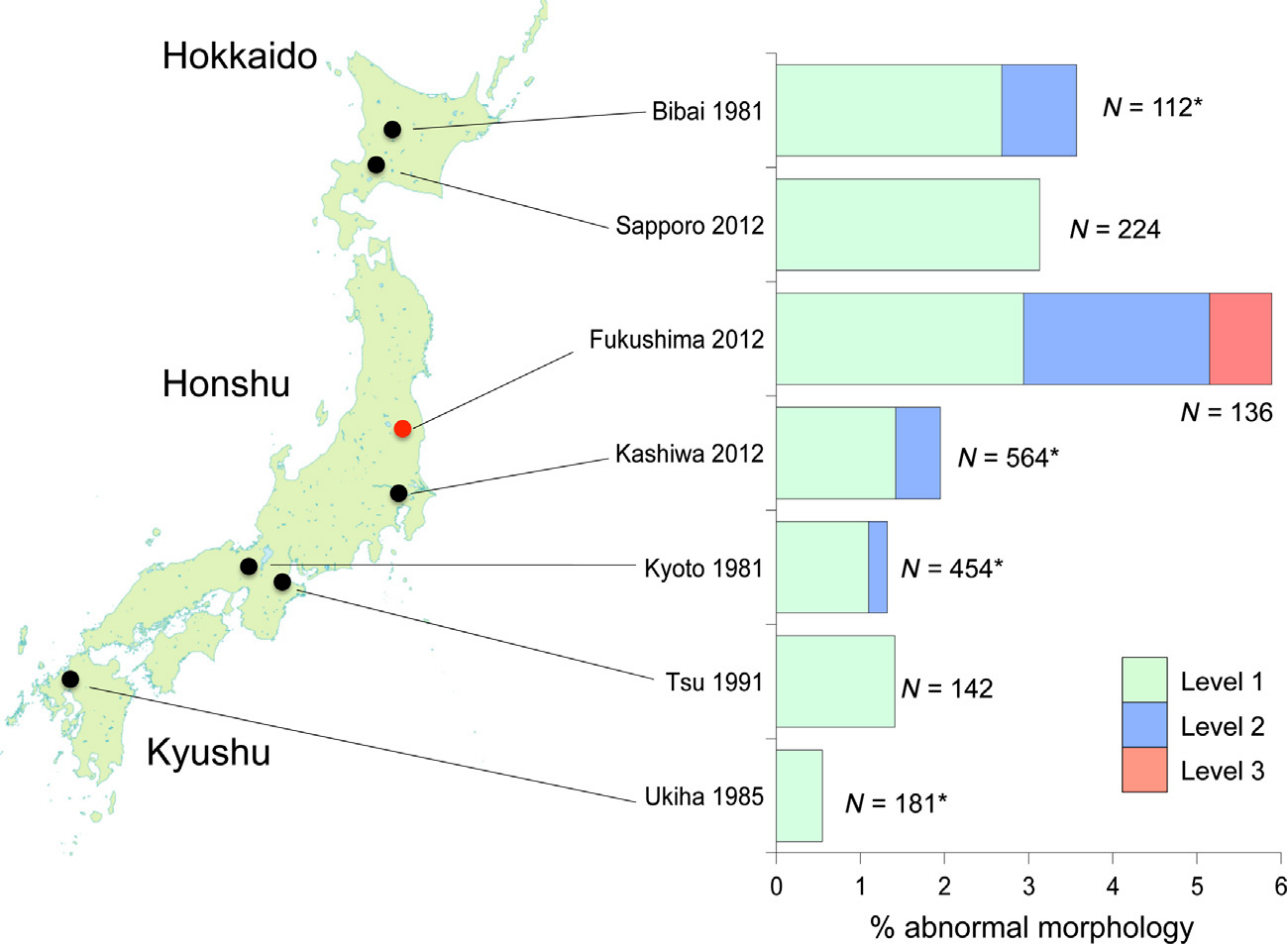
Percentage abnormal morphologies in *T. nigriabdominalis* first-instar gall formers from seven populations. Asterisks in sample sizes indicate that first instars were collected from buds, whereas nonasterisked figures indicate that first-instars' cast-off skins were collected from galls. The definitions of three levels of abnormality are the same as in Fig. 1.

The first instars of *T. nigriabdominalis* collected in Fukushima exhibited a higher proportion of abnormalities (5.9%) than those in the six control areas (Fig. 6). However, in *T. nigriabdominalis,* the absolute number of abnormal individuals was small, so a significant difference was limited among the populations after the sequential Bonferroni correction (for Fukushima vs. Ukiha, log-likelihood ratio test, df = 1, χ^2^ = 8.6, *P *<* *0.05; for Fukushima vs. Kyoto, df = 1, χ^2^ = 7.7, *P *<* *0.05; for the other combinations, *P *>* *0.05). Nevertheless, a level-3 abnormality was found in only 1 first instar from Fukushima. This first instar lost 1 hind tibia, 1 mid-tibia, and 1 antenna because of necrosis during embryogenesis, but successfully molted into the second stadium (Fig. 4B).

The larval mortality of *T. nigriabdominalis* in Fukushima was compared with that in Sapporo, Hokkaido (Fig. S4B). Galls from Fukushima were collected from 1 elm tree in 3 June 2012, while those from Sapporo were collected from about 20 trees in 5 June 2012. In *T. nigriabdominalis* as well, mortality in the Fukushima population (16.9%) was significantly higher than that in the Sapporo population (1.3%) (log-likelihood ratio test, χ^2^ = 31.3, *P *<* *0.0001).

In *T. radicicola*, for which only three galls were collected, the gall formers exhibited abnormalities in all of the galls. One gall former was found dead at the first stadium in an incipient gall, one exhibited necrosis in three legs at the third stadium, and one lost a mid-tibia at the second stadium.

### Comparison between the 2012 and 2013 samples from Fukushima

Of 50 *T. sorini* and 347 *T. nigriabdominalis* gall formers collected in 2013*,* first instars with abnormal morphology accounted for 6.0% and 2.6%, respectively, and all of these first instars were categorized in level 1, except 1 *T. sorini* that was at level 2. In the comparison between the years, gall formers of *T. sorini* and *T. nigriabdominalis* were categorized into three groups; healthy, morphologically abnormal, and dead aphids. In this categorization, a gall former with abnormal morphology at the first stadium was considered dead if it finally failed to grow in the gall. Thus, the abnormal category includes living aphids only, and in this category, aphids that had lost appendages accounted for 85.7% and 88.2% in *T. sorini* and *T. nigriabdominalis,* respectively. When the galls were collected in the Fukushima area on 21 May 2013, the radiation dose was 2.4 *μ*Sv/h at 1 m and 4.0 *μ*Sv/h at ground level.

**Figure 7:**
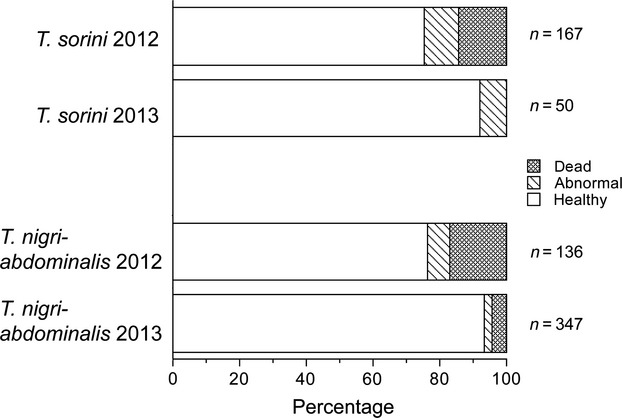
Comparison of the fates of gall formers collected from one elm tree in the Fukushima area between 2012 and 2013. Gall formers in the galls were categorized into three groups: healthy, morphological abnormal, and dead aphids (fundatrices).

The proportions of dead and abnormal aphids decreased, and the proportion of healthy aphids increased in 2013 compared to the 2012 samples in both species (Fig. 7). The mortality of *T. sorini* decreased from 14.4% to 0.0%, and similarly, the mortality of *T. nigriabdominalis* declined from 16.9% to 4.3%. Logistic regression indicated that the fates of gall formers significantly changed between the years (df = 2, χ^2^ = 33.1, *P *<* *0.0001) and between the species (df = 2, χ^2^ = 8.9, *P *<* *0.011). However, there was no significant interaction between years and species (df = 2, χ^2^ = 4.4, *P *=* *0.111). These results suggest that the healthiness of the two *Tetraneura* species was much improved from 2012 to 2013 in the Fukushima population.

## Discussion

Because the present study is based on aphid samples collected from only one host tree near Fukushima Daiichi, there is as yet no proof that morphological abnormalities in aphids are increasing or prevailing in the Fukushima area. However, investigations on organisms just after the Nuclear Power Plant accident are almost completely lacking, so that the results from two aphid species may provide useful information for future studies including genetic one.

Morphological comparisons of *T. soniri* first instars reveal that the Fukushima population contains a higher percentage of morphological abnormalities than do control populations. Some kinds of abnormalities are not specific to Fukushima but are common to the control populations. Possibly, morphological abnormalities recorded in this study result from environmental stresses as well as genetic factors. For example, embryogenesis under poor nutritional conditions, or in low or high temperatures, or pathogens may be responsible for aberrant morphogenesis in aphids (Shingleton et al. 2003; Kobayashi and Murai 2012). In laboratory experiments, the application of precocene derivatives reportedly can induce abnormal phenotypes in several species of aphids (Hardie 1986). Furthermore, as a genetic factor, inbreeding may contribute to the emergence of abnormalities. Eriosomatine aphids, including *Tetraneura* species, have a breeding system conducive to inbreeding (Dixon 1998; Akimoto 2006), so that homozygosity of deleterious recessive genes due to inbreeding may be causally linked to the high incidence of morphological abnormalities. Nevertheless, the Fukushima samples notably contained not only many aberrant first instars but also five highly deformed first instars of *T. sorini* and *T. nigriabdominalis*. To my knowledge, such deformations have not previously been reported in aphids. Furthermore, the finding that three *Tetraneura* species on the same tree commonly exhibit abnormalities at high percentages suggests that in Fukushima, some extrinsic factors are responsible for the induction of morphological abnormalities.

Akimoto (2006) examined the phenotypic effect of intense inbreeding on first-instar aphids using the eriosomatine aphid *Prociphilus oriens*. Enforced intraclonal mating (genetically identical to selfing) resulted in lowered hatchability and delayed timing of egg hatch compared to first instars derived from outbreeding. Morphologically, selfing often led to enlarged appendages relative to body size in the first instars, thus resulting in alterations in the allometric relationships among body parts. However, selfing did not lead to malformations in terms of the asymmetry of bilateral characters, appearance of novel characters, or extreme dwarfism. The involvement of inbreeding in the emergence of malformations is evident in some cases (Olsson et al. 1996; McCune et al. 2002), but not in others (Williams et al. 2008). This discrepancy may reflect a different extent of inbreeding in wild populations. Where an organism has a regular system of partial inbreeding, deleterious genes with a serious phenotypic effect would be readily purged from the population. As a result, inbreeding would not lead to morphological abnormalities but to a fitness reduction (i.e., inbreeding depression) due to slightly deleterious mutations (DeRose and Roff 1999; Hedric and Kalinowski 2000; Frankham et al. 2007). Therefore, it is reasonable to suppose that frequent occurrences of abnormalities in Fukushima are affected by some extrinsic factors rather than inbreeding. Except for the Fukushima area, the only example of malformation was detected in Bibai, Hokkaido in 1987. The egg of this individual was produced in the autumn of 1986, when Hokkaido was slightly affected by fallout from the Chernobyl nuclear accident (Fukuda 2007). However, it is not clear whether this accident is linked to the emergence of the malformation.

Comparison with a Hokkaido population suggests that gall formers of *T. sorini* and *T. nigriabdominalis* in Fukushima more often fail to develop in the galls. Such high mortality during development might be due to the resistance of the host plant to gall induction rather than extrinsic factors such as mutagens. A number of studies have documented that the performance or viability of gall-forming insects is reduced when they are associated with host plants of the resistant type, which coexists with the susceptible type in a population (Anderson et al. 1989; Simms and Fritz 1990; Paige and Capman 1993). However, the fact that the growth performance of gall formers did not vary between the Fukushima and Iwamizawa populations (Fig. S5) implies that the quality of the host plant in Fukushima was not inferior to that in Iwamizawa. Thus, the results of this analysis suggest that a high mortality in Fukushima cannot be attributed to host unsuitability, but may possibly be caused by external factors.

Environmental contaminants or other extrinsic factors may lead to genomic alterations that ultimately cause malformations (Gilbert and Epel 2008). Mutagens that impede the developmental process of *Tetraneura* aphids might include chemical substances such as ethyl methane sulfate (EMS) and N-methyl-N'-nitro-N-nitrosoguanidine (MNNG) (Singer and Kusmierek 1982) or radioactive substances (Møller and Mousseau 2006). Because the present study focuses on morphology, it is not possible to clarify which mutagen is linked to the morphological abnormalities. Nevertheless, it is difficult to imagine the occurrence of chemical mutagens around the study area in Fukushima. The study area is surrounded by rice and other crop fields and natural forests, and there is no industrial waste disposal plant in the vicinity. This collection locality is in a planned evacuation area from which local people are obliged to evacuate, and thus, cultivations in 2011 and 2012 were completely abandoned. As a result, no chemical substances including insecticides and herbicides have been applied. An increasing number of studies have documented morphological abnormalities from wild anurans (Stocum 2000; Lannoo 2008), and several alternative hypotheses are proposed for the cause, with difficulty determining the critical factor. In the case of *Tetraneura* aphids as well, several factors may be associated with the incidence of abnormality and mortality. However, because abnormalities in *Tetraneura* are detected in the first-instar gall formers, causal factors should be one that affected embryogenesis in eggs. In this respect, predators and parasites are safely ignored as the causal factor.

Analysis of the 2013 Fukushima samples corroborated the view that morphological abnormality and high mortality in the 2012 samples cannot be attributed to chemical mutagens, plant factors, nor inbreeding. This is because the healthiness of gall formers of the two species is improved on the same host tree in 2013 despite the lack of environmental changes. This result implies that some extrinsic factors that appeared specifically from 2011 to the spring 2012 may have led to frequent morphological abnormality and mortality.

The study area in Fukushima was intensively contaminated with radioactive substances emitted from the Fukushima Daiichi Nuclear Power Plant. At the time of the nuclear accident, *Tetraneura* aphids were overwintering in the egg stage on the bark surface of elm trunks, and thus, the eggs were exposed directly to the fallout. A report from the Nuclear Regulation Authority, Government of Japan (2011b) indicates that weeds collected in the same place as the study area (Yamakiya, Kawamata Town) on 16 March 2011 were contaminated with iodine-131 at 727,000 Bq/kg, cesium-134 at 157,000 Bq/kg, and cesium-137 at 158,000 Bq/kg. The eggs were probably exposed to the same level of radiation. In early May, first-instar gall formers hatched and started gall formation under intense contamination. In mid-June, second-generation winged adults migrated from the cracked galls to gramineous plants, and subsequent generations continued to reproduce asexually on the roots. The soil surface was also contaminated with radionuclides (Tanaka et al. 2013a), so that *Tetraneura* aphids infesting the host roots, near the soil surface or 5 cm deep at the most, may also have been exposed to external radiation. Thus, if radiation damaged the genes of the developing embryos, such damaged genes may have been inherited through clonal lines to the autumnal sexual generation.

In the autumn of 2011, winged adults returned to elm trees from gramineous plants and produced the sexual generation, whose females produced eggs after copulation. Thus, damaged genes, if any, might have been genetically recombined during sexual reproduction, resulting in a large variation in the accumulation of damaged genes among first instars hatching from the eggs in 2012. In addition to the accumulation effects of deleterious genes, eggs deposited in the autumn of 2011 may have been subjected to radiation on the elm bark for approximately 4 months. The bark surface of trees reportedly was intensively contaminated with radionuclides (Kuroda et al. 2013; Tanaka 2013). Therefore, if radiation was the main factor causing abnormal morphologies, then two mechanisms can be assumed: (1) the accumulation of damaged genes through clonal reproduction (Muller's ratchet) and the release of diverse phenotypes due to the first recombination after the accident, and (2) short-term physiological effects due to continuous exposure to radiation on the host bark during embryogenesis (Møller et al. 2005; Mousseau and Møller 2012b). In some insect species, released radioactive substances have been attributed to malformations (Hesse-Honegger and Wallimann 2008; Hiyama et al. 2012). The proportion of abnormalities detected in Fukushima was probably underestimated because seriously deformed first instars were selected out before gall formation due to low viability (Møller 1997) or intraspecific competition (Akimoto and Yamaguchi 1997).

In gamma irradiation experiments conducted so far, study insects were subjected to various doses of radiation ranging from dozens to hundreds of Gy to examine the impact on insect survival, development, and reproduction (Cole et al. 1959; Elbadry 1965; Elvin et al. 1966; Burgess and Bennett 1971; Burditt et al. 1989). Under such experimental conditions, most dividing cells in the insects were possibly adversely affected over the entire body. By contrast, in the Fukushima area, the environmental radioactivity level (4–6 *μ*Gy/h) was much lower than that used in the irradiation experiments. However, in the radiation-contaminated area, radioactive particles were scattered over the soil surface or the bark surface of host trees (Kuroda et al. 2013; Tanaka 2013; Tanaka et al. 2013a). Thus, we have to consider the effects of radioactive particles on the surface of oviposition substrates. My main hypothesis is that if a radioactive particle was on the egg surface or in the vicinity on the bark surface, radiation from the particle possibly affected a restricted number of cells during embryogenesis. Thus, depending on the distance from contamination spots on the bark surface, eggs may have been subjected to varying levels of radiation. In addition, I speculate that as the eggs of *Tetraneura* aphids are smaller than 1 mm in the major axis, not only gamma radiation but also alpha and beta radiation from hot particles may have had strong effects on embryogenesis. On the other hand, contaminated food cannot be considered a cause of malformations because the overwintering egg does not ingest food or water.

The malformed individual with a bifurcated abdomen (Fig. 2) successfully reached adulthood and developed embryos in her abdomen. This result implies that even if external radiation was responsible for this malformation, some cells were detrimentally affected during embryogenesis, but other cells were free from the adverse effects of radiation. Similarly, the low level of abnormality in the second generations indicates that radionuclides may not have penetrated a closed gall, which develops from one point of the leaf tissue through the rapid proliferation of cells. In fact, translocation of radiocesium from a contaminated branch to new leaves growing after the accident was not clearly observed (Tanaka et al. 2013b). There may be other reasons for the lack of abnormalities in the second generation. Galls were induced on leaves, on average, a few meters from the trunk, and the embryogenesis of the second generation is temporally separated from that of the first generation. These spatial and temporal differences may partly be responsible for the lack of abnormalities in the second generation. Gamma radiation may easily penetrate gall wall and affect the second-generation aphids inside. However, the gall wall may prevent alpha and beta radiation, which, in contrast, probably affects embryogenesis directly on the bark. Otherwise, the difference in the incidence of abnormalities between the first and second generations may be explained by differences in the penetrance of damaged genes between generations.

I assume two possibilities for the improvements in viability and healthiness in 2013. First, a decrease in radiation dose around the study tree may be responsible for the improvements, although a level of 4 *μ*Gy/h is still observed at the ground. The second possibility is the action of natural selection for radiation tolerance in the aphid species. Through 2011 and 2012, I suppose that aphid clones were subjected to radiation on the host plants and that many first instars were selected out before and during gall formation. Thus, natural selection may have favored aphid clones tolerant to radiation. If this is true, the populations of the two *Tetraneura* species may have survived the first impact of fallout by acquiring radiation tolerance. High reproductive rates in aphids may contribute to this improved viability. On the other hand, it is reported that radiation may interact with other environmental stressors such as drought or high temperature and create negative effects in some years (Mousseau et al. 2013). However, there is no record of special climatic events, for example, drought and extreme low or high temperatures, during the period of gall formation in May 2011 and 2012 in the Fukushima area (Japan Metrological Agency; Fukushima WMO Station http://www.data.jma.go.jp/obd/stats/etrn/view/monthly_s3_en.php?block_no=47595%view=1). Therefore, it appears to me that morphological abnormality and mortality are temporary phenomena just after nuclear fallout and disappear in later generations.

## Conclusion

Several factors are probably responsible for morphological abnormality and mortality in insects, and at present, there is no decisive evidence that any single factor is causally related to the observed abnormalities. However, comparisons between the research results in 2012 and 2013 suggest that a specific environmental factor from 2011 until the spring 2012 was involved in the incidence of abnormalities and mortalities. Evidence from this study suggests that of several potential factors, radioactive fallout from Fukushima Daiichi in the spring 2011 is most likely to have had the strongest effect, because other factors can be readily ignored as the causal factor.

Provided that radioactive fallout is the critical factor, I further postulate that morphological abnormalities found in first-instar gall formers result from the localized effect of radioactive particles on developing embryos in eggs. At present, it is difficult to determine which of the two hypotheses (the accumulation of damaged genes and short-term physiological effects) adequately accounts for the occurrence of morphological abnormalities in the gall formers. However, because most of the abnormalities were not inherited by the next generation or by the next year's generations, the possible effect of radioactive particles appears to have been confined to short-term physiological effects arising from, for example, oxidative stress (Mousseau and Møller 2012b). However, because damaged genes possibly just start to accumulate and because contamination continues long, the adverse effects of damaged genes may appear some years later. Thus, it is necessary to survey the effects of radiation on the morphology and genetics of *Tetraneura* aphids continuously over several years.

This study possibly demonstrates the operation of selective pressures on radiation tolerance just after nuclear fallout. This kind of selective death has not been reported in the studies of the wildlife in Chernobyl. Through natural selection for aphid clones tolerant to radiation, coupled with a lowered level of environmental radiation, *Tetraneura* populations in Fukushima are apparently recovering. However, it is not clear whether this tendency continues hereafter or not. Therefore, in the future study, it is critical to explore whether the aphid populations overcome the nuclear contamination through acquiring radiation tolerance or confront abnormalities again several years later due to the accumulation of damaged genes.

## Supporting Information

Figure S1 Gall formation by *Tetraneura* aphids.

Figure S2 Level-1 morphological abnormalities in *Tetraneura* first-instar gall formers.

Figure S3 Level-1 tissue necrosis and level-2 appendage loss.

Figure S4 Percentage mortality in *T. sorini* and *T. nigriabdominalis* gall formers in their gall.

Figure S5 Relationship between first-instar hind femur length and adult hind femur length in the same individual.
