# Supplementary figures and images for: Morphological abnormalities in gall-forming aphids in a radiation-contaminated area near Fukushima Daiichi: selective impact of fallout?
Source: Ecol Evol. 2014 Jan 13;4(4):355–69. doi: 10.1002/ece3.949 (PMC3936383; doi:10.1002/ece3.949)

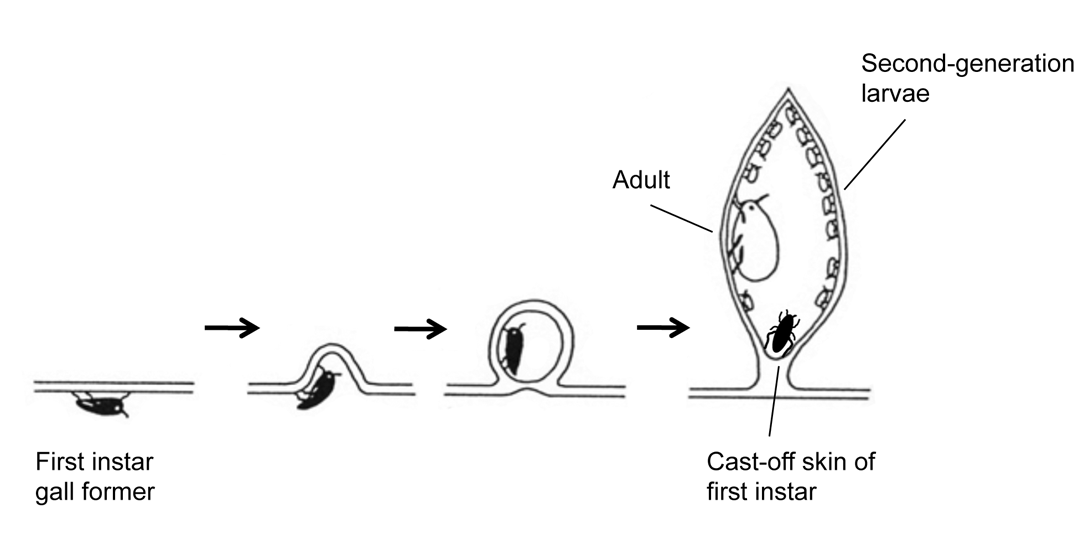

Supplement: Figure S1 — Gall formation by Tetraneura aphids. [file ece30004-0355-sd1.tif]

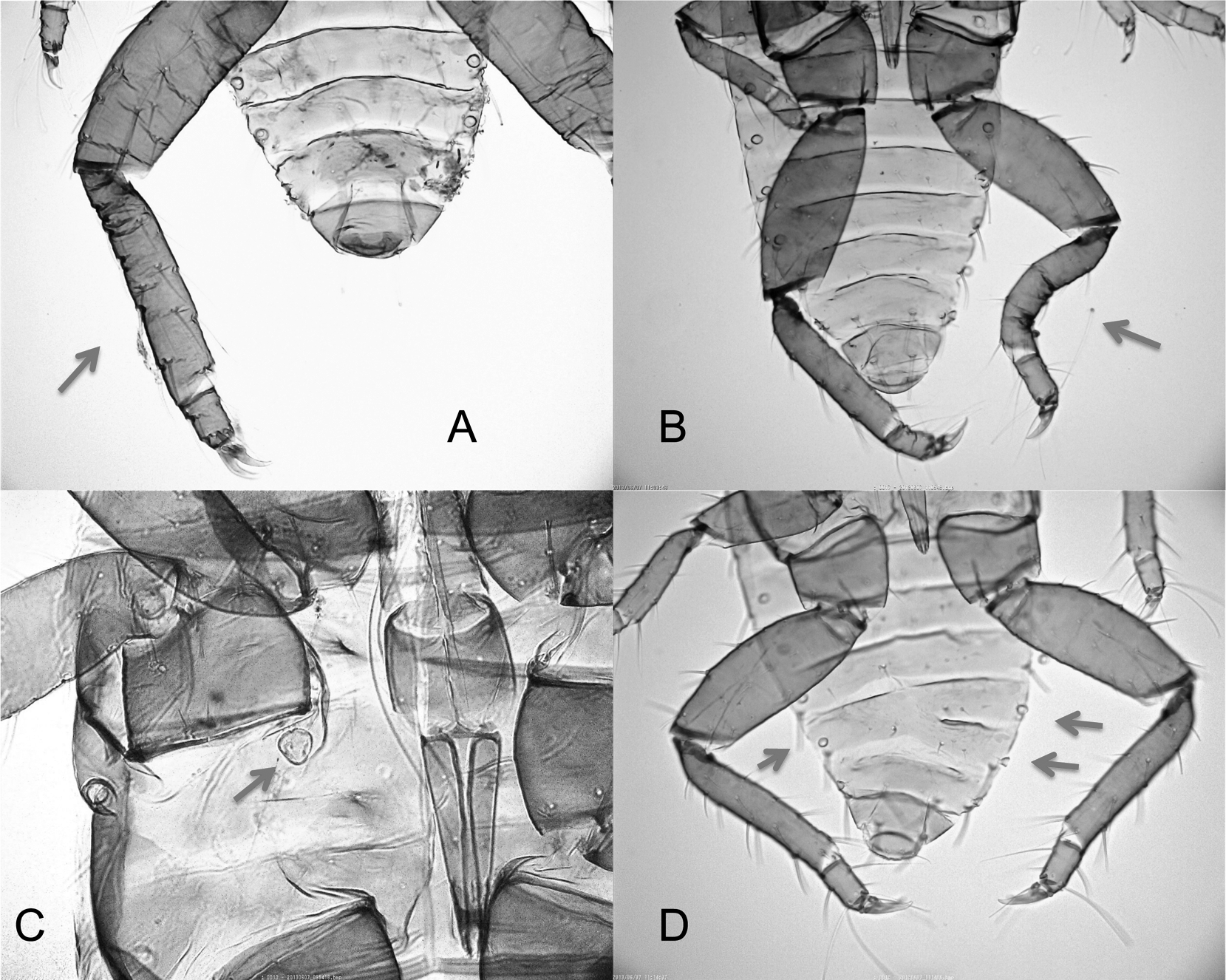

Supplement: Figure S2 — Level-1 morphological abnormalities in Tetraneura first-instar gall formers. [file ece30004-0355-sd2.tif]

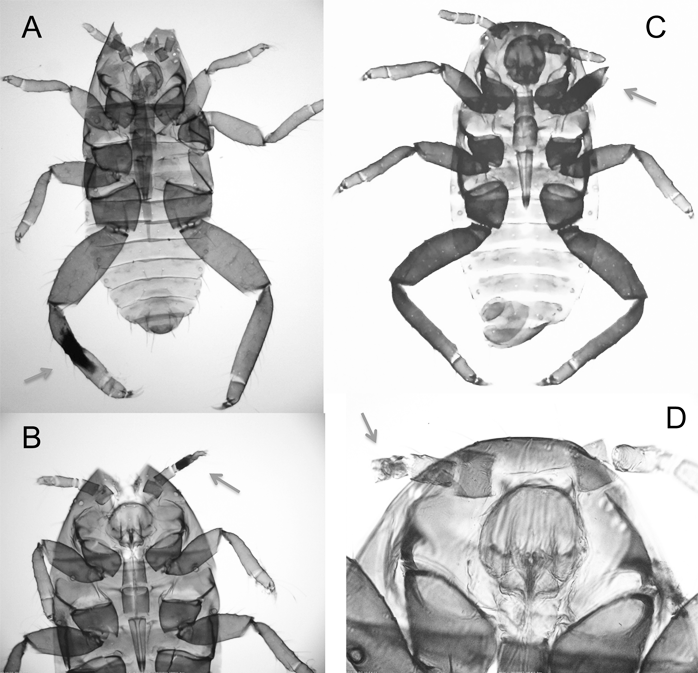

Supplement: Figure S3 — Level-1 tissue necrosis and level-2 appendage loss. [file ece30004-0355-sd3.tif]

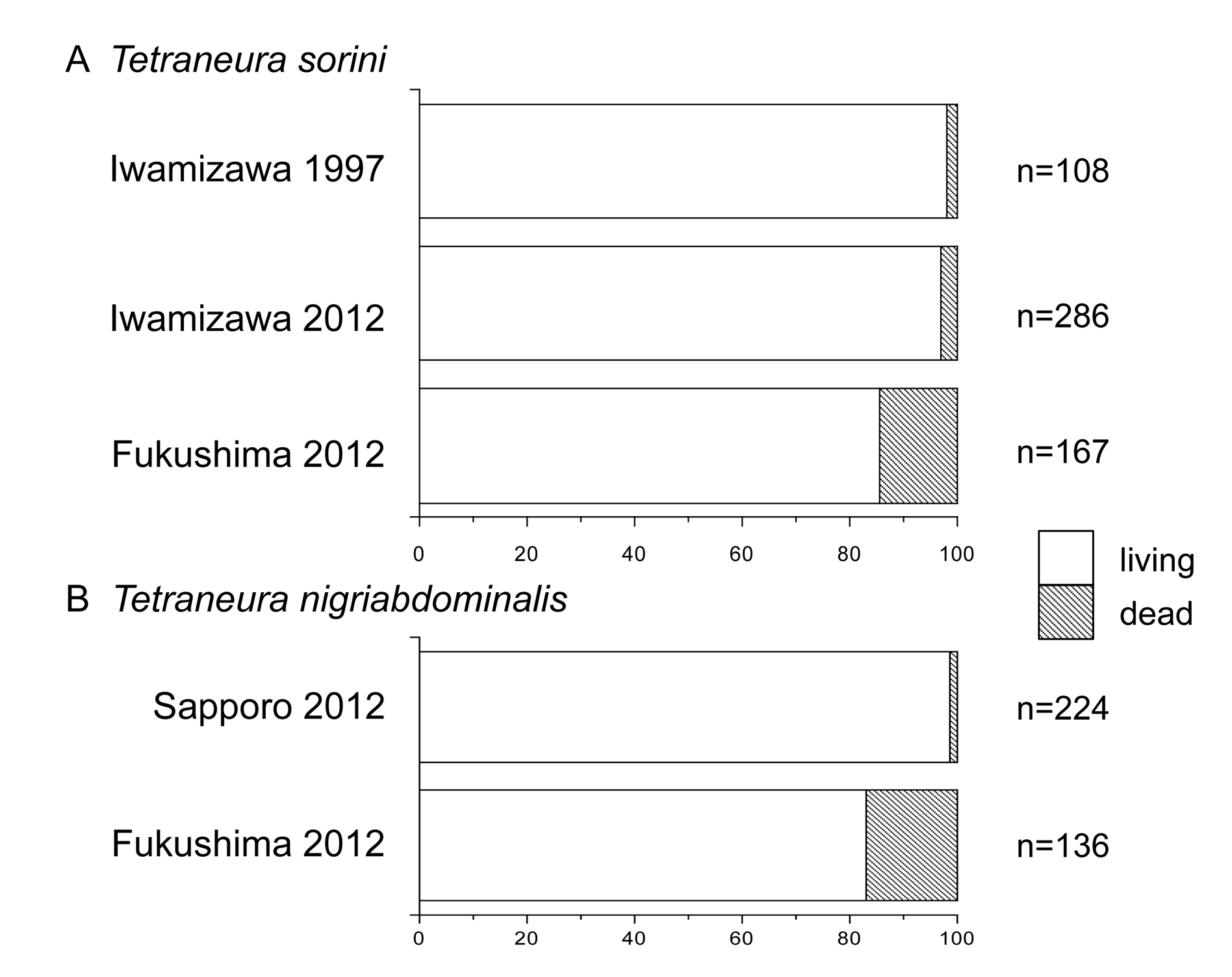

Supplement: Figure S4 — Percentage mortality in T. sorini and T. nigriabdominalis gall formers in their gall. [file ece30004-0355-sd4.tif]

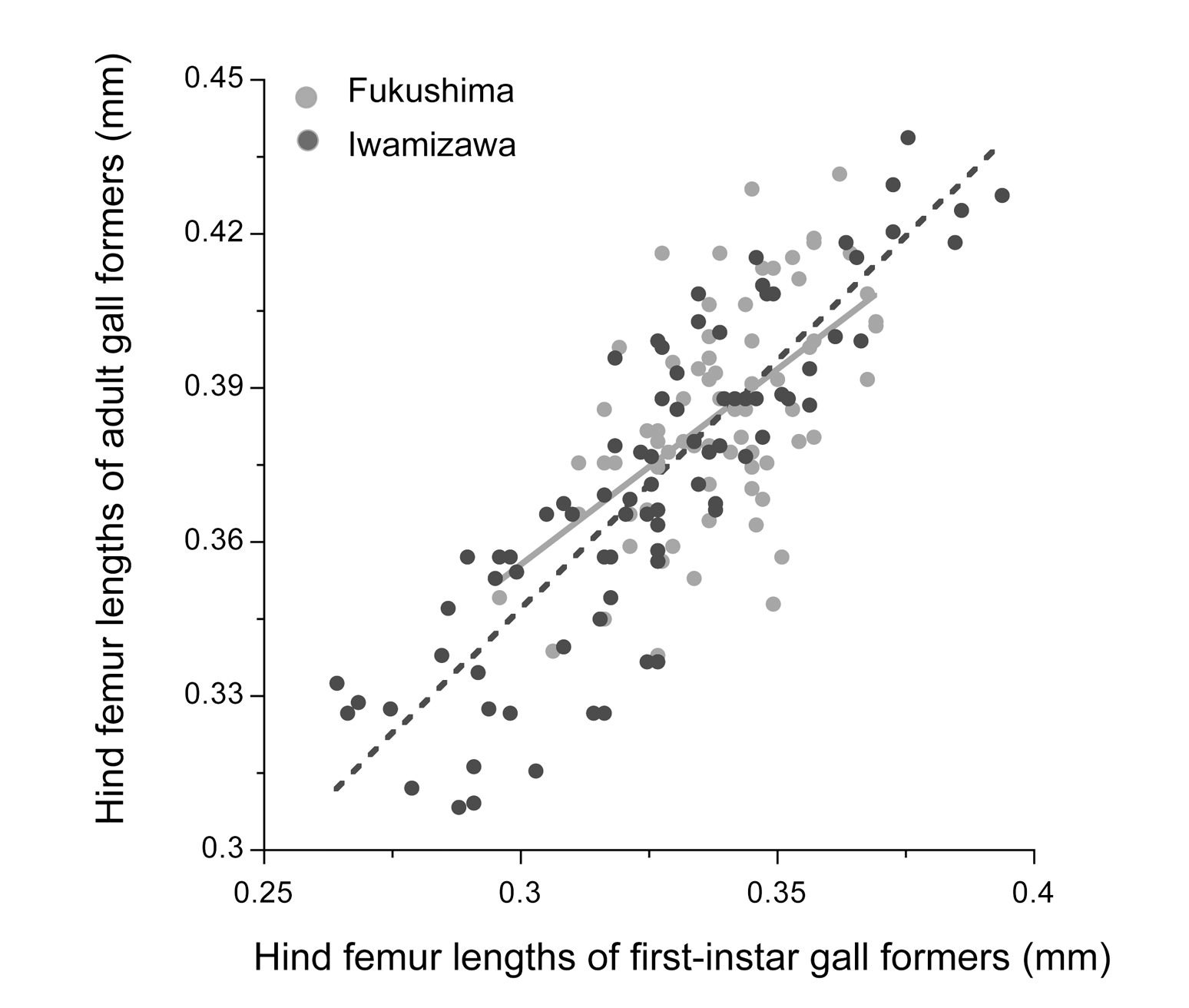

Supplement: Figure S5 — Relationship between first-instar hind femur length and adult hind femur length in the same individual. [file ece30004-0355-sd5.tif]
